# Perioperative Outcomes of Noncardiac Surgical and Interventional Procedures in Adults with Single-Ventricle Physiology: A Retrospective Cohort Study

**DOI:** 10.3390/jcm15134921

**Published:** 2026-06-24

**Authors:** Montserrat Ribas-Ball, Laura González, Ekaterine Popova, Clara Bordes, Patricia Galan, Laura Villarino, Alfons Gómez, Maria Josefa Azpiroz, Marcos de Miguel, Laura Dos-Subirà, Miriam de Nadal

**Affiliations:** 1 Department of Anaesthesiology and Intensive Care, Hospital Vall d’Hebron, Passeig de la Vall d’Hebron, 119-129, 08035 Barcelona, Spain; 2 Department of Surgery and Morphological Sciences, Faculty of Medicine, Universitat Autònoma de Barcelona, Passeig de la Vall d’Hebron, 119-129, 08035 Barcelona, Spain; 3 Centro de Investigación Biomédica en Red de Enfermedades Cardiovasculares, Instituto de Salud Carlos III, 28029 Madrid, Spain; 4 Department of Cardiology, Hospital Vall d’Hebron, Passeig de la Vall d’Hebron, 119-129, 08035 Barcelona, Spain

**Keywords:** single-ventricle physiology, noncardiac surgery, adult congenital heart disease, perioperative complications, Fontan circulation

## Abstract

**Background/Objectives:** Adults with single-ventricle physiology (SVP) represent a growing population with complex cardiovascular conditions and an increasing need for noncardiac surgical and interventional procedures. However, perioperative outcomes in this group remain poorly characterized. This study aimed to provide a descriptive characteristic of perioperative management, complications and mortality in adults with SVP undergoing noncardiac surgical and interventional procedures. **Methods:** We conducted a retrospective cohort study including all adult patients (≥18 years) with SVP who underwent noncardiac surgical and interventional procedures requiring anesthesia or sedation at a tertiary university hospital between 1 January 1995 and 30 November 2023. Demographic data, comorbidities, type of procedure and anesthetic technique were collected. Complications were defined as intraoperative or postoperative adverse events requiring intervention or associated with hemodynamic, respiratory, or cardiovascular instability. Primary outcomes were perioperative complications and all-cause mortality at 24 h, 30 days, and one year, with mortality reported at the patient level. **Results:** A total of 114 procedures were performed in 67 patients (mean age 32.3 ± 10.8 years). Most procedures were elective (78.9%) and minimally invasive, frequently performed under sedation, with or without local anesthesia (67.5%). Common comorbidities included arrhythmias (46.3%), liver disease (49.3%), and heart failure (17.9%). The overall complication rate was 6.1% (2.6% intraoperative, 3.5% postoperative). Mortality was 1.5% in 24 h, 2.9% in 30 days and 5.9% at one year. Most clinically relevant adverse events occurred in patients with earlier-stage palliation, advanced functional limitation or multiple comorbidities. **Conclusions:** Perioperative outcomes in adults with SVP undergoing noncardiac surgical and interventional procedures were acceptable when procedures were elective and managed in specialized settings. Risk remains heterogeneous and appears to be influenced by physiological status and stage of palliation.

## 1. Introduction

Single-ventricle physiology (SVP) includes a heterogeneous group of congenital heart defects in which only one functional ventricular chamber supports both systemic and pulmonary circulations, making biventricular repair impossible [1,2,3,4,5]. Adults with SVP often develop long-term complications such as arrhythmias, thromboembolism, heart failure, liver disease, and reduced functional capacity, resulting in significant morbidity throughout life [6,7,8,9,10,11].

Advances in pediatric cardiology, cardiothoracic surgery and perioperative care have resulted in a growing adult population living with complex congenital heart disease (CHD), who now represent most of the CHD population [12,13,14]. With the aging of adults with CHD, the need for noncardiac surgical and interventional procedures is increasing due to age-related comorbidities, specific heart-defect conditions, arrhythmias, gastrointestinal disorders, trauma and malignancy. Among this growing population, SVP represents one of the most complex forms of CHD, often associated with specific clinical challenges. In this context, adults with SVP may require interventions including abdominal, orthopedic, hepatobiliary, electrophysiologic and endoscopic procedures [15,16,17,18,19,20]. Recent expert consensus statements, including a contemporary European multidisciplinary document in which our research group has participated, further emphasize that adults with CHD undergoing noncardiac procedures represent a growing and high-risk population requiring specialized multidisciplinary care [21].

Anesthetic management in SVP patients is particularly challenging due to the wide variability in hemodynamic conditions across this population. Hemodynamic profiles differ substantially according to anatomical substrate, stage of palliation (if any), ventricular function and the presence of complications related to structural pathology or previous surgeries. In consequence the impact of ventilation strategies, intrathoracic pressure, systemic and pulmonary vascular resistances and rhythm stability is highly context-specific reflecting the considerable variability in anesthetic conditions across patients with SVP [22,23,24]. Chronic cyanosis and multi-organ involvement further increase perioperative vulnerability [25,26,27]. Large database studies in adults with CHD have shown increased perioperative morbidity and mortality compared with non-CHD patients, with complex lesions, including single ventricle, representing one of the highest-risk subgroups [10,28]. This increased risk has also been emphasized in the 2022 European Society of Cardiology (ESC) Guidelines [29] on cardiovascular assessment and management of patients undergoing NCS, as well as in a recent multidisciplinary statement providing specific anesthetic practical recommendations for this population [21].

Despite these risks, perioperative outcomes of adult SVP patients undergoing noncardiac surgical and interventional procedures remain underreported and most available evidence comes from pediatric cohorts, mixed adult CHD populations, or isolated case reports [11,12,13,20,21,22,23,24,25]. Although recent consensus statements provide practical recommendations for perioperative care, they also underscore the limited high-quality evidence specifically addressing adults with SVP undergoing noncardiac procedures [21]. Therefore, adults with SVP represent an underrepresented population in perioperative research, with gaps in knowledge regarding perioperative management, complication rates, mortality and optimal anesthetic management.

The aim of this study was to provide a descriptive characterization of perioperative management, complications and mortality associated with noncardiac surgical and interventional procedures in adult patients with SVP requiring anesthesia.

## 2. Materials and Methods

### 2.1. Study Design and Setting

We conducted a retrospective cohort study at a tertiary university hospital, including all adult patients (≥18 years) with a diagnosis of SVP (either palliated or not), who underwent noncardiac surgical or interventional procedures requiring anesthesia or sedation between 1 January 1995 and 30 November 2023.

Participants were identified through institutional electronic medical records and surgical and anesthesia databases. We excluded patients with incomplete clinical data, those without a confirmed SVP diagnosis and procedures not requiring anesthesia or sedation. When patients underwent multiple eligible procedures, each procedure was included as a separate perioperative event. Given the descriptive nature of the study and the limited number of adverse outcomes, formal clustering-adjusted analyses were not performed. All statistical analyses were performed using IBM SPSS Statistics for Windows, Version 30.0 (IBM Corp., Armonk, NY, USA).

This study did not require trial registration due to its retrospective observational design. We followed the STROBE reporting guidelines to ensure transparent reporting of our findings (Supplementary Table S1).

### 2.2. Ethics

The study was approved by local ethics committees on 21 April 2023 (Protocol ID PR(AG)128/2023) and conducted in accordance with the Declaration of Helsinki.

### 2.3. Study Management

Each procedure was considered an independent event. Patient age, clinical status and functional capacity were recorded at the time of each intervention. The data collection encompassed both paper and electronic hospital records. Demographic variables, comorbidities, and relevant medical history were obtained from the medical charts. SVP was defined as any anatomical defect preventing separation of pulmonary and systemic circulations. Cardiac surgical palliation, when present, was documented, and functional status was classified according to the New York Heart Association (NYHA) system.

A noncardiac procedure was defined as any surgical or interventional procedure requiring anesthesia or sedation in which the primary indication was not the treatment of structural heart defects. Surgical procedures were classified into low, intermediate, and high risk according to the 2022 European Society of Cardiology guidelines for cardiovascular assessment in noncardiac surgery [29].

Anesthetic management was adjusted to each patient, covering everything from procedural sedation to general anesthesia, according to the definitions established by the American Society of Anesthesiologists (ASA). Moderate sedation (conscious sedation) was defined as a drug-induced depression of consciousness where patients responded purposefully to verbal commands, with preserved airway and cardiovascular functions. Deep sedation/analgesia was defined as a drug-induced depression of consciousness where patients could not be easily aroused but respond to repeated or painful stimulation; airway and ventilatory functions could be impaired and might have required assistance, although cardiovascular function was usually preserved. Both moderate and deep sedation were administered in those procedures in which sedation was the anesthetic technique of choice. For intravenous sedation, institutional protocols were followed. Sedation was typically achieved using midazolam (0.02–0.05 mg/kg IV), propofol (0.5–1 mg/kg IV), and/or remifentanil (0.025–0.1 μg/kg/min), according to patient characteristics, procedural requirements and anesthesiologist discretion. All medications were standard hospital formulations routinely available at Hospital Vall d’Hebron (Barcelona, Spain).

General anesthesia was defined as a drug-induced loss of consciousness in which patients were not arousable even to painful stimulation, and both ventilatory and cardiovascular functions could be impaired, often requiring support. Regional anesthesia included neuraxial blocks (intradural and epidural blocks). Local anesthesia and peripheral nerve blocks were techniques that were always combined with general anesthesia or sedation.

### 2.4. Study Endpoint

The primary objective of this study was to provide a descriptive characterization of perioperative management and outcomes in adult patients with SVP undergoing noncardiac surgical or interventional procedures requiring anesthesia.

Specifically, we assessed the incidence of intraoperative and postoperative complications, defined as any adverse clinical event occurring during or after the procedure that required intervention or was associated with hemodynamic, respiratory or cardiovascular instability. Intraoperative complications included events such as hemodynamic instability, arrhythmias and cardiorespiratory arrest. Postoperative complications included arrhythmias, bleeding, infection and transient hemodynamic or rhythm abnormalities. We also evaluated all-cause mortality at 24 h, 30 days and one year following each procedure, reported at the patient level. These outcomes were selected to provide insight into perioperative risk and clinical outcomes in this underrepresented and high-risk adult population.

### 2.5. Sample Size

The study size was determined by the total number of eligible cases available during the study period. Given the retrospective design and the scarcity of SVP in the adult population, no formal sample size calculation was performed. All eligible cases were included to maximize statistical power and cohort representativeness.

### 2.6. Statistical Analysis

Continuous variables were summarized using measures of central tendency and dispersion (mean ± standard deviation or median with interquartile range, as appropriate), while categorical variables were expressed as frequencies and percentages. Normality was assessed based on data distribution and sample size. Given the descriptive nature of the study and the limited number of adverse outcomes, analyses were restricted to descriptive statistics. Patient-level characteristics were summarized using the total number of patients as the denominator, whereas procedure-related variables and perioperative complications were summarized using the total number of procedures. When patients underwent multiple procedures during the study period, each procedure was included as a separate perioperative event. Because observations were not fully independent and the number of adverse outcomes was low, no inferential analyses, regression models, or clustering-adjusted statistical approaches were performed. Mortality at 24 h, 30 days and one year was summarized at the patient level using frequencies and percentages. No time-to-event or survival curve analyses were performed.

## 3. Results

We included 114 NCSs performed on 67 patients between 1 January 1995 and 30 November 2023. The mean patient age was 32.3 ± 10.8 years, and 35 patients (52.2%) were female. The most prevalent underlying CHDs were double-inlet left ventricle (DILV) in 25 patients (37.3%) and tricuspid atresia in 17 patients (25.4%). The remaining 25 patients (37.3%) presented a heterogeneous group of CHDs, including valvular atresia/stenosis, hypoplastic left ventricle, double-inlet/outlet ventricles and other associated complex congenital lesions. Baseline patient-level characteristics, including NYHA functional class, oxygen saturation, comorbidities and prior cardiac surgeries, are summarized in Table 1.

In our cohort, arrhythmia represented the most frequent comorbidity and occurred in 31 patients (46.3%), followed by liver disease in 33 patients (49.3%) and heart failure in 12 patients (17.9%). Other comorbidities were less frequent. Regarding baseline clinical status, most patients were in NYHA functional Class II (28; 42.0%), followed by Class I (19; 28.6%), while 14.3% and 15.1% were in Classes III and IV. The mean baseline oxygen saturation was 89.2 ± 8.7, and the mean number of prior cardiac surgical procedures per patient was 2.4 ± 1.5.

At the time of surgery, most procedures were performed in patients with advanced stages of palliation. Specifically, eight procedures (7.0%) were carried out in non-palliated patients, 15 (13.2%) after Stage I palliation, including 13 with a systemic-to-pulmonary shunt and two with pulmonary artery banding, and 11 (9.6%) after Stage II palliation (Glenn procedure). Most procedures (67; 58.7%) were performed in patients with complete Fontan circulation (FC), while the remaining 13 procedures (11.4%) were undertaken in patients who had undergone other palliative or percutaneous interventions.

The distribution of surgical procedures according to specialty is shown in Table 2. Of the 114 procedures, 46 (40.4%) involved cardiology-related interventions mainly catheter ablation and cardioversion. Digestive endoscopic procedures accounted for 22 intervention (19.3%), predominantly gastroscopies, followed by angiological (11.4%) gynecological (11.4%) and general surgical procedures (7%) (Table 2).

According to the 2022 ESC guidelines for cardiovascular assessment and management of patients undergoing noncardiac surgery [29], most procedures were classified as low surgical risk (64.0%), followed by intermediate-risk (24.6%) and high-risk procedures (8.8%) (Supplementary Table S2).

Procedure-related characteristics are summarized in Table 3. Most procedures were elective 90 (78.9%), whereas 24 (21.1%) were urgent. Regarding admission status, 25 procedures (21.9%) were managed on an outpatient basis, while 55 (48.2%) required admission to a general ward and 34 (29.8%) required critical care admission. Sedation with or without local anesthesia was the most frequently used anesthetic approach, accounting for 77 procedures (67.5%). General anesthesia was used in 31 procedures (27.2%), whereas other anesthetic techniques accounted for the remaining six procedures (5.3%). All procedures were performed by anesthesiologists.

We observed complications in seven procedures (6.1%), including three intraoperative (2.6%) and four postoperative (3.5%) events. Six of these procedures were elective, and one was urgent. All complications occurred in patients aged 20 to 53 years, with NYHA functional classes between II and IV. Among the three intraoperative complications, two were major adverse events. Cardiac arrest occurred during peritoneal dialysis catheter placement in a patient with Stage I palliation and NYHA IV, resulting in death within 24 h. Respiratory arrest during arrhythmia ablation in a patient with Stage I palliation and NYHA II required endotracheal intubation with complete recovery within hours. The third intraoperative event was a self-limited skin reaction during angiological intervention in patients with Fontan circulation and NYHA II. Postoperative complications included arrhythmia recurrence with heart failure, post-procedural bleeding after colonoscopy, transient fever following an angiological procedure and transient asymptomatic bradycardia following intrauterine device placement. The latter two events were minor, self-limited and did not require specific treatment.

Mortality was analyzed at the patient level and reported at 24 h, 30 days and one year after surgery. Cumulative mortality was 1.5% (1/67 patients) in 24 h, 2.9% (2/67 patients) in 30 days and 5.9% (4/67 patients) in one year (Table 4). Only one patient (1.5%), who was in NYHA Class IV, with stage I palliation and multiple comorbidities including severe pulmonary hypertension and a history of stroke, died within the first 24 h of the postoperative period. Overall, four patients died within one year after surgery.

**Figure 1:**
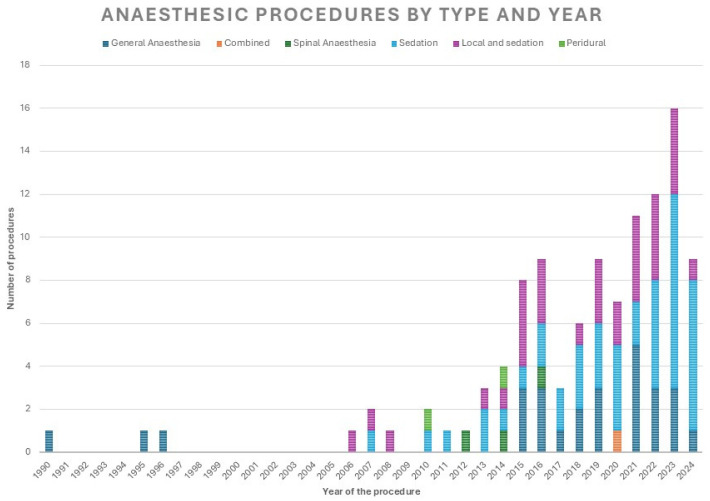
Distribution of noncardiac surgery types in adult patients with single-ventricle physiology.

All observed adverse outcomes occurred with early-stage palliation, higher NYHA functional class, and the presence of multiple comorbidities. Given the limited number of events, no formal comparisons between subgroups were made. Over the study period we observed a progressive increase in the number of surgical procedures, as well as the rising use of general anesthesia in more-recent years (Figure 1), reflecting changes in perioperative practice and the increasing number of adults living with single-ventricle physiology.

## 4. Discussion

Adult patients with SVP undergoing noncardiac surgical and interventional procedures represent a particularly challenging population due to their complex circulation and the long-term consequences of CHD [1,2,3,4,5,10,11,12]. In our cohort of 114 procedures performed on 67 adult patients over nearly three decades, the main finding is that perioperative morbidity (6.1%) and short-term mortality (2.9% at 30 days) were relatively low, even when considering all reported events, including some that were minor or of limited clinical significance, despite the high prevalence of comorbidities such as arrhythmias (46.3%), liver disease (49.3%) and heart failure (17.9%). The long study period (1995–2023) spans major advances in anesthetic management, congenital heart disease surgery and intensive care strategies. Although we observed temporal changes in procedural volume and anesthetic practice, a formal stratified analysis by historical era was not performed due to the limited sample size, low event rate and procedural heterogeneity. Consequently, any era-specific comparisons would have been difficult to interpret reliably. Importantly, most procedures were classified as low surgical risk according to ESC 2022 criteria [29] (64.0%), with only a small proportion of high-risk interventions (8.8%), which likely contributed to the favorable overall outcomes observed. A substantial proportion of procedures in our cohort consisted of minimally invasive interventions, primarily cardiology-related procedures such as catheter ablations and cardioversions, as well as digestive endoscopic procedures. Together, these procedures accounted for nearly 60% of all interventions and were commonly performed under sedation in routine clinical practice. This procedural distribution should be considered when interpreting the overall low complication rates observed in our study and may limit the generalizability of our findings to patients undergoing morecomplex noncardiac surgical procedures. If minor or self-limited events are considered separately, the impact of clinically relevant mortality would appear even lower, further supporting the overall safety observed in this cohort. Despite these concerns, most procedures in our cohort were elective (78.9%) and carried out under sedation, with or without local anesthesia (67.5%). This more conservative anesthetic approach may have helped maintain overall circulatory stability, as it is likely reduced sudden hemodynamic changes that can be poorly tolerated in patients with SVP [4,10,25]. Recent multidisciplinary recommendations, including the ESC-supported consensus statement on anesthetic management in adults with CHD, also emphasize individualized anesthetic strategies and careful tailoring of perioperative management in this population [21]. We observed a gradual increase in the use of general anesthesia in recent years, which probably reflects the growing complexity of the procedures performed, as well as increasing confidence and experience in managing these patients within a specialized center.

Importantly, adverse events were not evenly distributed across our cohort. The only death within the first 24 h occurred in a patient with Stage I palliation, NYHA Class IV, severe pulmonary hypertension and multiple comorbidities. This finding reinforces that patients in earlier palliative stages or those with advanced functional limitation remain particularly vulnerable [17,18,19,22]. It also highlights the importance of careful preoperative functional assessment and individualized risk evaluation.

More than half of the procedures were performed in patients with completed Fontan circulation (58.7%). Although Fontan physiology generally provides greater circulatory stability than that in earlier stages, it is associated with progressive long-term complications, including arrhythmia, liver disease, thromboembolic events and heart failure [9,16,18,19,26]. The high prevalence of arrhythmia (46.3%) and liver disease (49.3%) observed in our cohort is consistent with these well-recognized long-term complications.

Most published adult series focus predominantly on patients with established Fontan circulation, whereas evidence regarding adults with non-Fontan or minimally palliated single-ventricle physiology remains scarce [9,18,19]. Although our cohort included patients across the spectrum of palliation, the limited sample size and low number of adverse events precluded robust subgroup analyses. Consequently, our findings should be interpreted primarily as descriptive and hypothesis-generating rather than comparative between physiological subgroups.

Systemic cyanosis is widely recognized as an important determinant of morbidity and perioperative risk in adults with single-ventricle physiology and other forms of complex CHD [3,10,18]. However, the available sample did not allow meaningful analyses according to cyanotic status or baseline oxygen saturation, highlighting the need for larger multicenter studies specifically designed to address this question.

The study by Buendía-Fuentes et al. [9] is particularly relevant when interpreting our results. In their multicenter cohort of adults with single-ventricle physiology without Fontan palliation, long-term mortality reached 22.6% and atrioventricular valve regurgitation, thrombocytopenia, renal dysfunction, and QRS prolongation were associated with worse outcomes. These findings underline the baseline fragility and heterogeneity of this population. In this context, the relatively low perioperative mortality observed in our study suggests that, in carefully selected patients, especially those with established Fontan circulation and reasonable functional status, noncardiac procedures can be undertaken with acceptable risk when managed appropriately.

Most available data on noncardiac procedures in SVP patients derive from pediatric populations [11,12,13,22]. Brown et al. [11] reported early postoperative adverse events in 11.8% of children with SVP undergoing noncardiac procedures. Similarly, data from the Pediatric Perioperative Cardiac Arrest (POCA) Registry demonstrated increased anesthesia-related cardiac arrest in children with CHD, with single-ventricle lesions disproportionately represented [12]. These reports have reinforced concerns regarding anesthesia exposure in this physiology. In contrast, our adult cohort demonstrated lower complication rates. This difference may reflect greater physiological stability among long-term survivors, more-conservative case selection, and care delivered within a specialized adult CHD center [3,20,28]. It may also reflect survival bias, as adult SVP patients represent a subset who have already survived earlier high-risk stages.

Regarding procedure profiles, cardiology-related interventions accounted for 40.4% of procedures, predominantly catheter ablation and cardioversion, reflecting the high burden of arrhythmias in this population [9,16,18]. Digestive endoscopic procedures were also common, particularly among Fontan patients, likely related to surveillance and management of Fontan-associated liver disease [16,18,27].

Over the study period, we also observed a progressive increase in the number of procedures performed. This finding reflects the expanding adult CHD population and the continued improvement in long-term survival [2,3,5,15]. As this population ages, the need for noncardiac interventions will continue to grow. International guidelines recommend that adults with complex CHD, particularly those with SVP, undergo surgical procedures in specialized centers with multidisciplinary expertise [3,20,28]. Our findings support this approach, as outcomes in this high-risk group were favorable when care was delivered within a structured ACHD program. Of note, there remains a clear lack of adult-specific perioperative data in SVP patients [22,23,24,25,28]. Most recommendations are extrapolated from pediatric experience or based on expert opinion [3,10]. The recent consensus statement [21] highlights this gap in high-quality evidence and provides practical recommendations derived mainly from expert consensus rather than robust prospective data, underscoring the need for real-world evidence in this field, which supports the relevance of our study. Therefore, our cohort provides real-world data that may help inform clinical decision-making and serve as a reference for other similar healthcare systems managing this population.

### Strengths and Limitations

Our study has several strengths. First, it focuses exclusively on adult patients with SVP undergoing noncardiac surgical and interventional procedures, a population that remains underrepresented in perioperative research. While the previous literature has primarily focused on pediatric populations or mixed cohorts of congenital heart disease, our study provides one of the largest contemporary descriptions of perioperative management and outcomes across the spectrum of adult SVP. Second, the long inclusion period (1995–2023) provides insights into the evolution of surgical practice and anesthetic strategies over time. Third, the inclusion of patients at different palliative stages, from non-palliative to Fontan full circulation, reflects real-world heterogeneity and enhances external validity. Fourth, the data on mortality at 24 h, 30 days, and one year provides better understanding of immediate and medium-term outcomes, which is particularly relevant in a population with progressive systemic complications. Finally, our study reflects the local experience of a tertiary university hospital for congenital heart disease, aligned with international recommendations, providing a practical model for structured perioperative management in complex congenital patients.

Our study also has several limitations. First, its retrospective, single-center design presents potential selection and information bias. Second, detailed intraoperative hemodynamic data and standardized perioperative management variables were not consistently or fully available across all cases, limiting the ability to identify specific modifiable risk factors. Third, although relatively large for this infrequent population, the sample size and the small number of events precluded meaningful comparative analyses between groups and limited the ability to identify independent risk factors. In addition, the heterogeneity of congenital diagnoses further complicates risk stratification, particularly comprising different stages of palliation. Fourth, most procedures were minor and elective, frequently performed under sedation-based anesthetic management. While this likely contributed to the low complication rate, it limits the generalizability of these findings to major or highly invasive surgeries. Fifth, given that some patients underwent multiple procedures during the study period, observations were not fully independent. This may have introduced within-subject correlation, potentially influenced procedure-based event rates and limited the validity of inferential statistical analyses. Furthermore, the small number of adverse events precluded the use of clustering-adjusted or hierarchical models. Therefore, results should be interpreted as descriptive rather than inferential. Finally, the extended inclusion period may also have introduced temporal heterogeneity in perioperative management and patient selection.

## 5. Conclusions

Adults with SVP undergoing noncardiac surgical and interventional procedures face complex cardiovascular challenges. However, in our cohort, most procedures were elective and minimally invasive, resulting in low perioperative complications and mortality. A higher proportion of adverse outcomes was observed in patients with early-stage palliation, advanced functional limitations, or multiple comorbidities. Anesthesiologists must also be aware of the significant anatomical variability and the different surgical stages these patients undergo throughout life, as each stage may require distinct anesthetic management strategies. These findings highlight that, with careful planning and specialized care, noncardiac surgical and interventional procedures can be performed safely in this growing adult population, while emphasizing the need for larger studies to better guide perioperative management.

## Figures and Tables

**Table 1:** Baseline patient-level characteristics (*N* = 67 patients).

Characteristic	Value
Age (years), mean ± SD	32.3 ± 10.8
Female sex, *n* (%)	35 (52.2)
Baseline oxygen saturation (%), mean ± SD	89.2 ± 8.7
Prior cardiac surgical procedures, mean ± SD	2.4 ± 1.5
**NYHA functional class, *n* (%)**	
Class I	19 (28.6)
Class II	28 (42.0)
Class III	10 (14.3)
Class IV	10 (15.1)
**Underlying congenital heart disease, *n* (%)**	
Tricuspid atresia	17 (25.4)
Double-inlet left ventricle (with/without TGA)	25 (37.3)
Other congenital heart diseases	25 (37.3)
**Comorbidities, *n* (%)**	
Arrhythmias	31 (46.3)
Liver disease	33 (49.3)
Heart failure	12 (17.9)

**Abbreviations**: TGA—transposition of the great arteries; NYHA—New York Heart Association; SD—standard deviation.

**Table 2:** Distribution of noncardiac surgical procedures by specialty (*N* = 114).

Surgical Specialty	*n* (%)
Cardiology	46 (40.4)
Digestive	22 (19.3)
Angiology	13 (11.4)
Gynecology	13 (11.4)
General surgery	8 (7.0)
Cardiac surgery	5 (4.4)
Other specialties *	5 (4.4)

* Other specialties include neurosurgery, dermatology, maxillofacial surgery, plastic surgery, and ophthalmology.

**Table 3:** Procedure-related characteristics (*N* = 114 procedures).

Characteristic	Value
**Procedure urgency *n* (%)**	
Elective	90 (78.9)
Urgent	24 (21.1)
**Admission status *n* (%)**	
Outpatient	25 (21.9)
General ward admission	55 (48.2)
Critical care admission	34 (29.8)
**Type of anesthesia *n* (%)**	
Sedation with or without local anesthesia	77 (67.5)
General anesthesia	31 (27.2)
Other	6 (5.3)

**Table 4:** Patient-level cumulative mortality (*N* = 67).

Timepoint	Mortality, *n* (%)
24 h	1 (1.5%)
30 days	2 (2.9%)
1 year	4 (5.9%)

## Data Availability

The data used in the present study is part of a larger dataset. The datasets generated and analyzed during the current study are available and can be supplied by the corresponding authors upon reasonable request. The data not used for this manuscript will be employed in future manuscripts.

## Supplementary Materials

The following supporting information can be downloaded at https://www.mdpi.com/article/10.3390/jcm15134921/s1; Table S1: STROBE statement—checklist of items that should be included in reports of cohort studies. Table S2: Distribution of procedures according to surgical risk (ESC 2022 classification).
